# The balance of Polo-like kinase 1 in tumorigenesis

**DOI:** 10.1186/1747-1028-4-4

**Published:** 2009-01-22

**Authors:** Lin-Yu Lu, Xiaochun Yu

**Affiliations:** 1 Division of Molecular Medicine and Genetics, Department of Internal Medicine, University of Michigan Medical School, 109 Zina Pitcher Place, BSRB 1520, Ann Arbor, Michigan, 48109, USA

## Abstract

Polo-like kinase 1 (Plk1) belongs to a family of conserved serine/threonine kinases with a polo-box domain, which have similar but non-overlapping functions in the cell cycle progression. Plk1 plays a key role to ensure the normal mitosis. Interestingly, overexpression of Plk1 is associated with tumor development and could serve as a prognostic marker for many cancers. Due to Plk1 overexpression, several Plk1 inhibitors have been developed and tested for the cancer treatment. However, in a recent study, it has been suggested that down-regulation of Plk1 could also induce aneuploidy and tumor formation *in vivo*. Therefore, a normal level of Plk1 is important for mitosis. And caution should be taken when Plk1 inhibitors are used in the clinical trial and their side effects including tumorigenesis should be carefully evaluated.

## Review

Polo-like kinases (Plks) are serine/threonine kinases with an N-terminal kinase domain and a C-terminal polo-box domain. The domain structure of this family is evolutionary conserved from yeast to mammals[1]. In mammals, there are four different Plks that all participate in the cell cycle regulation [2]. Plk1 is the best characterized member in this group. Numerous lines of evidence demonstrate that Plk1 plays critical roles in multiple stages of mitosis (reviewed in [3]). Either up-regulated or down-regulated Plk1 could induce mitotic defects that result in aneuploidy and tumorigenesis. Therefore, balanced Plk1 levels are important for normal mitosis. In this review, we will summarize current understandings of the role of Plk1 in mitosis, with emphasis on its association with tumorigenesis.

## Multiple roles of Plk1 in mitotic progression

Plkl functions in almost every stage of mitosis, as evidenced by changing of its localization during mitotic progression. Plk1 localizes at the centrosomes during inter-phase and prophase, and is found at kinetochores in pre-metaphase and metaphase. It later relocates to spindles during anaphase and finally resides at the midbody during telophase [4-7]. This dynamic localization of Plk1 correlates with several distinct functions of Plk1 that are through its different substrates recognition and phosphorylation during mitosis. Plk1 directly promotes mitotic entry by activating Cdc25C and Cdk1/Cyclin B complex [8-10]. It contributes to centrosome maturation and drives microtubule nucleation through phosphorylating Nlp, Kizuna and Asp [11-14]. Plk1 facilitates kinetochore assembly and potentially regulates of spindle assembly checkpoint by interaction with INCENP, Bub1 and BubR1 [5,15-17]. Plk1 is also indispensable for the completion of cytokinesis by regulating Ect2 and RhoA [18,19]. Consistent with the critical role of Plk1 in mitosis, Plk1-deficient mice are early embryonic lethal. Plk1-null embryos fail to pass 8-cell stage and no mitotic cells were observed in these embryos [20]. Although Plk1-null zygotes could go through three rounds of mitosis and develop to 8-cell stage, it is most likely that maternal Plk1 protein and mRNA are still sufficient for a few rounds of mitosis. Once depleting the maternal material contributed Plk1 protein, cell cycle is arrested.

## Plk1 overexpression is associated with tumorigenesis

Proper cell cycle progression is critical for maintaining genomic stability. Mitosis is particularly tightly regulated as deregulated mitosis would lead to improper segregation of chromosomes. Checkpoints including G2/M checkpoint, kinetochore and spindle checkpoint have therefore evolved to ensure proper onset of mitosis and correct transmission of genetic material to daughter cells [21]. The mitotic progression is mainly promoted by cyclin-dependent kinases and further controlled by several critical mitotic kinases including Plk1 [22,23]. Therefore, the expression level of these mitotic kinases must be tightly regulated. Overexpression of these kinases can override those mitotic checkpoints and lead to immature cell division without proper chromosome alignment and segregation, which will result in aneuploidy, one of the major causes for tumorigenesis [24,25]. Because of this, these mitotic kinases including Plk1 are often considered as proto-oncogenes, whose overexpression is often observed in tumor cells [26]. Meanwhile, aneuploidy and tumorigenesis can also result from centrosome abnormality, particularly centrosome amplification defects [27,28]. Centrosome duplication and maturation regulated by Plk1 occurs from late S phase to prophase [11]. Abnormal centrosome amplification may lead to multipolar spindles and results in unequal segregation of chromosomes [29]. Thus, Plk1 overexpression may also increase the centrosome size and/or centrosome number, which will also lead to improper segregation of chromosomes, aneuploidy, and tumorigenesis.

To date, overexpression of Plk1 has been observed in a number of human cancers including non-small-cell lung cancer [30], head and neck cancer [31], esophageal cancer [32,33], gastric cancer [32,34,35], melanomas [36,37], breast cancer[38], ovarian cancer [39], endometrial cancer [40], colorectal cancer [41,42], glioma [43], papillary carcinoma [44], pancreatic cancer [45,46], prostate cancer [47], hepatoma [48], leukemia and lymphoma [49,50], bladder cancer [51], and thyroid cancer [52]. Many of these studies have demonstrated that Plk1 overexpression correlates with tumor progression and patient survival rate in a variety of cancers [30-33,35-37,42,48-50]. Therefore, Plk1 is proposed as a prognostic marker for human cancers.

Although Plk1 is often overexpressed in human cancers, the *Plk1 *gene is rarely amplified, indicating that transcriptional or post-transcriptional regulation of Plk1 are affected in cancer cells. The association of Plk1 overexpression with cancers could be explained as a result of high mitotic index of tumor cells since Plk1 levels are cell cycle-regulated with a peak during mitosis [4,7]. Indeed, early studies of Plk1 in different fetal and adult tissues have shown that Plk1 levels are much higher in thymus, spleen and testis, which have more proliferating cells [53-55]. However, Plk1 overexpression has been indicated as the cause of tumor formation instead of being the consequence of high mitotic index during tumor cell proliferation. Overexpression of Plk1 in NIH3T3 fibroblasts transformed the cells into oncogenic foci in soft agar and more importantly lead to tumor formation when injected into nude mice [56].

## Development of Plk1 inhibitors

Since Plk1 is considered as a "proto-oncogene", inhibition of Plk1 could be an effective treatment for cancers. Several strategies of inhibiting Plk1 activity have thus been tested in cancer therapeutic trials. One of the strategies is to deplete the expression of Plk1 by using anti-sense oligonucleotides (ASO) or small interfering RNA (siRNA) to block Plk1 translation or transcription. This rationale comes from the effect of Plk1 depletion in cell cultures, which leads to mitotic arrest and apoptosis of the cells [57,58]. To date, several groups have also shown successful suppression of tumor growth by this approach *in vivo*. For example, intravenous injection of Plk1 ASO significantly suppressed growth of A549 cells in tumor xenografts [59]. Similarly, Intravesical administration of Plk1 siRNA suppressed bladder cancer growth in an orthotopic bladder cancer mouse model [60]. Plasmid-based U6 promoter-driven short hairpin RNA has also been demonstrated to be effective in suppressing growth of HeLa S3 xenografts [61]. However, due to the intrinsic problems including dose-limiting side effects, inadequate penetration into the tumor tissues, and degradation by endogenous nucleases, it is difficult to achieve a consistently high efficacy [62,63]. Optimization of the delivery system is ongoing, for example using ASO-loaded HSA nanoparticles [64].

Using small molecules to inhibit Plk1 is another approach, as these molecules are easier to be delivered into cells and are less likely to be degraded. For most chemical inhibitors, they are designed to suppress important functional domains. For Plk1, one important domain is the serine/threonine kinase domain at the N-terminus. Same as other serine/threonine kinases, the activity of Plk1 kinase domain requires ATP. Thus, ATP analogues have been designed and screened for inhibiting Plk1. The first identified inhibitor of this kind is scytonemin isolated from cyanobacteria with an IC_50 _of 2 μM [65]. Recently, a much more specific ATP competitor, BI 2536, has been identified. It inhibits the kinase activity of Plk1 with an IC_50 _of only 0.83 nM. More importantly, this molecule displayed 1000-fold selectivity for Plk1 over 63 other kinases tested [66,67]. BI 2526 not only induced mitotic arrest and apoptosis in a number of tumor cells, but also suppressed the growth of human tumor xenografts in nude mice [66]. Currently, a phase I clinical trial is ongoing with the treatment of advanced solid tumors [68]. Besides BI 2526, several other compounds including thiophene benzimidazole compound 1 and pyrimidine derivative DAP-81 have also been shown to specifically inhibit Plk1 activity [69,70].

Besides ATP analogs, several non-ATP competitors were identified to block substrates binding to Plk1 kinase domain. For example, ON01910 inhibits Plk1 effectively with an IC_50 _of 9–10 nM [71]. This drug potently induced apoptosis in 151 tumor cell lines tested and inhibited tumor growth of three human tumor xenografts in nude mice. Currently, this drug is under evaluation in phase I clinical trials [72]. Similarly, a non-ATP competitive inhibitor TAL was recently identified that specifically inhibits Plk1 over 93 other kinases with an IC_50 _of around 19 nM [73]. Using a structural-guided approach, a non-ATP competitive inhibitor cyclapolin 1 was also identified to inhibit Plk1 with an IC_50 _of 20 nM [74].

Except the kinase domain, the other important domain of Plk1 is the C-terminal polo-box domain (PBD). This domain is a phospho-amino acid binding motif [10,75,76] that is essential for the substrate recognition and localization of Plk1 to centrosomes, kinetochores, and the midbody [77,78]. Chemical compounds that block the PBD would thus block the functions of Plk1. One advantage is that the PBD is not present in kinases other than Plks. Therefore, it is unlikely that small molecules targeting the PBD will affect other kinases. Based on this hypothesis, the natural product thymoquinone and the synthetic thymoquinone derivative Poloxin were identified that inhibited Plk1 by interfering the PBD [79]. Although with less potency, these drugs provided the proof of the principle that targeting PBD is a promising approach.

One concern for all Plk1 inhibitors is that they inhibit other Plks with similar potencies, most likely due to the structural similarities between these four family members. For example, besides inhibiting Plk1 with IC_50 _of 0.83 nM, BI 2536 also inhibit Plk2 and Plk3 with IC_50 _3.5 nM and 9 nM respectively [66]. Such broad inhibition of all Plks may have unexpected side effects as different Plks have none overlapping roles in mitosis, and not all Plks are overexpressed in cancers [80,81].

## Loss of Plk1 also leads to tumor formation

Paradoxically, loss of Plk1 is also associated with tumor formation. Several missense mutations of Plk1 have been identified in different cancer cell lines with various tissue origins [82]. These mutations locate at the C-terminal PBD and suppress the interaction between Plk1 and HSP90, a molecular chaperon that functions in protein folding [83]. As a result, mutant Plk1 is unstable without the chaperon, which leads to low expression levels of Plk1 in these cell lines. Since Plk1 is a key mitotic kinase, the reduction of Plk1 may also induce mitotic defects that lead to aneuploidy and tumorigenesis. This possibility has been proven by a recent *in vivo *study [20]. In this study, Plk1 heterozygous mice have been generated, in which the expression level of Plk1 was significantly decreased. A cohort of Plk1 wild type and heterozygous mice were maintained and the tumor development was monitored. Surprisingly, 30% of Plk1 heterozygous mice developed tumors at an average of 13–14 months old, and this was a 3-fold increase of tumor incidence compared with that in wild type mice. The major tumor type is lymphoma, probably due to the rapid proliferation of lymphocytes and the requirement for high expression of Plk1 to maintain the cell proliferation [55]. Moreover, correlated with the tumor phenotype, analysis of pre-malignant splenocytes displayed elevated aneuploidy in Plk1 heterozygous mice. Considering the importance of Plk1 in every stage of mitosis, reduced Plk1 levels might not only delay centrosome maturation and mitotic entry but also cause improper segregation of chromosomes and arrest cytokinesis. These events will eventually lead to aneuploidy and tumorigenesis. Thus, Plk1 can also be considered as a "haploinsufficient tumor repressor".

The tumorigenesis induced by either overexpression or down-regulation of Plk1 suggests the level of Plk1 has to be tightly regulated, which reflects its critical role in mitotic progression. Excessive Plk1 will override the mitotic checkpoint, amplify the centrosome abnormally, and chromosomes will segregate without proper alignment or unequally. Insufficient Plk1 will also leads to mitotic delay and improper separation of chromosome. In both scenarios, aneuploidy and tumors will occur. Similar associations of tumorigenesis with aberrant expression of other Plks and even other mitotic kinases were also observed [26]. For example, the expression of Plk3 is down-regulated in several human cancers [84-86] and *Plk3 *is therefore considered as a tumor-suppressor gene. Consistently, Plk3 knockout mice are viable and develop tumors in various organs in advanced stage [87]. *Plk4 *is another potential tumor suppressing gene residing at chromosome 4q28 in human, a region where loss of heterozygosity (LOH) is common in hepatoma samples [88]. Plk4-null mice are embryonic lethal and Plk4 heterozygous mice display 15-fold increase in incidence of liver and lung cancers than that of wild type mice [89]. These all suggest that the level of these kinases needs to be tightly regulated.

Like Plk1, Aurora A, is another multi-functional kinase involved in all stages of mitosis [90,91]. Overexpression of Aurora A has been reported in a number of cancers [92], and chemical compounds have been developed to target Aurora A in cancer therapeutic trials. Similar as Plk1, overexpression of Aurora A suppresses the mitotic checkpoint, induces various mitotic defects and transformation of fibroblasts both *in vitro *and *in vivo*. Moreover, like Plk1-null mice, Aurora A knockout mice fail to survive after blastocyst stage [93,94]. More importantly, Aurora A heterozygous mice with reduced Aurora A level are also tumor prone with a significant increased incidence of lymphoma. And lymphocytes from Aurora A heterozygous mice display elevated aneuploidy [93]. The similar phenotypes between Plk1 and Aurora A knockout mice suggest that these two mitotic kinases work in a similar pathway. Indeed, recent reports suggest that Plk1 is actually activated by Aurora A and its cofactor Bora before it activates CDK1 and promotes mitotic entry [95,96]. Again, the similar tumor phenotypes of Plk1 and Aurora A heterozygous mice suggest that balanced mitotic kinases are important for maintaining genomic stability. Reducing the levels of these mitotic kinases long term may induce tumorigenesis. Since a few drugs targeting Plks and Aurora kinases are currently in the clinical trails for cancer treatment, long term side effects including secondary tumor formation should be carefully evaluated.

## Concluding remarks

Plk1 is a critical kinase with multiple functions during mitotic progression. Its expression level has to be precisely regulated. Either overexpression or down-regulation of Plk1 will lead to tumorigenesis. Considering several ongoing clinical trials using Plk1 inhibitors, the side effects of these drugs in inducing genomic instability should be carefully observed.

## Competing interests

The authors declare that they have no competing interests.

## Authors' contributions

LL and XY contributed to the discussion and preparation of this manuscript. Both the authors read and approved the final manuscript.
